# A Rare Case of Acute Pancreatitis Due to Very Severe Hypertriglyceridemia (>10 000 mg/dL) Successfully Resolved With Insulin Therapy Alone: A Case Report and Literature Review

**DOI:** 10.1177/2324709618798399

**Published:** 2018-09-01

**Authors:** Vijay Gayam, Amrendra Kumar Mandal, Arshpal Gill, Mazin Khalid, Ruby Sangha, Mowyad Khalid, Pavani Garlapati, Bikash Bhattarai

**Affiliations:** 1 Interfaith Medical Center, New York, NY, USA; 2 Detroit Medical Center, Wayne State University, MI, USA

**Keywords:** hypertriglyceridemia, acute pancreatitis

## Abstract

A 48-year-old male presented to the psychiatric emergency room for dysmorphic mood. He was admitted to medical service for the management of hyponatremia, which was discovered in his initial laboratory workup. After the first day of admission, he developed abdominal pain and fever, and subsequent laboratory work revealed a triglyceride level of 10 612 mg/dL (reference range = 0-194 mg/dL). Computed tomography scan of the abdomen and pelvis revealed a hypodense lesion in the pancreas surrounded by a moderate amount of peripancreatic fluid suggestive of hemorrhagic pancreatitis. Based on the laboratory findings and imaging, we diagnosed acute pancreatitis (AP) secondary to hypertriglyceridemia. The patient was initiated on intravenous fluids and insulin to help decrease the triglyceride level with the plan to initiate apheresis. However, the patient improved on insulin therapy alone, which negated the need for apheresis, and the patient was discharged with fenofibrate with no further complications. While elevated triglycerides are a well-known cause of AP, we sought to assess various treatment options in management, especially considering a severely elevated triglyceride level of >10 000 mg/dL. Along with supportive care in AP, there are additional options in hypertriglyceridemia AP, including heparin, insulin, apheresis, antioxidants, and fibrates. Currently, there are no clear guidelines favoring one therapeutic option over the other.

## Introduction

Acute pancreatitis (AP) is a serious gastrointestinal disorder with a wide array of etiologies. The diagnosis of AP requires 2 of the following 3 features: (1) abdominal pain characteristic of AP, (2) serum amylase and/or lipase ⩾3 times the upper limit of normal, and (3) characteristic ﬁndings of AP on imaging, particularly computed tomography (CT) scan.^1^ The clinical severity of AP is stratiﬁed into 3 categories according to the revised Atlanta classification 2012: mild (no organ failure), moderately severe (transient organ failure <48 hours), and severe (persistent organ failure >48 hours). The treatment of AP consists of fluid resuscitation, pain management, and nutritional support.^1^ Hypertriglyceridemia (HTG) is a well-established etiology of AP. AP typically occurs with high levels of triglycerides (TGs), of at least 1000 mg/dL. The management of HTG-induced AP is usually supportive care. Insulin or apheresis may be given to help lower HTG. In this article, we report a case of a patient who developed AP secondary to very severe HTG (>10 000) successfully treated with insulin therapy.

## Case Report

A 48-year-old male presented to the psychiatric emergency room with dysmorphic mood. He was subsequently referred to medical service for the management of hyponatremia. His past medical history was notable for lumbar spondylosis managed with intermittent nonsteroidal anti-inflammatory drug use. The patient reported occasional alcohol consumption, with no intake during the past 4 weeks. He denied any history of diabetes or prediabetes, obesity, binge drinking, abdominal trauma, any offending drugs, and any procedures including endoscopic retrograde cholangiopancreatography. Family history was not significant for coronary artery disease, cerebrovascular accident, diabetes, dyslipidemia, pancreatitis, or gallstones.

**Figure 1.**
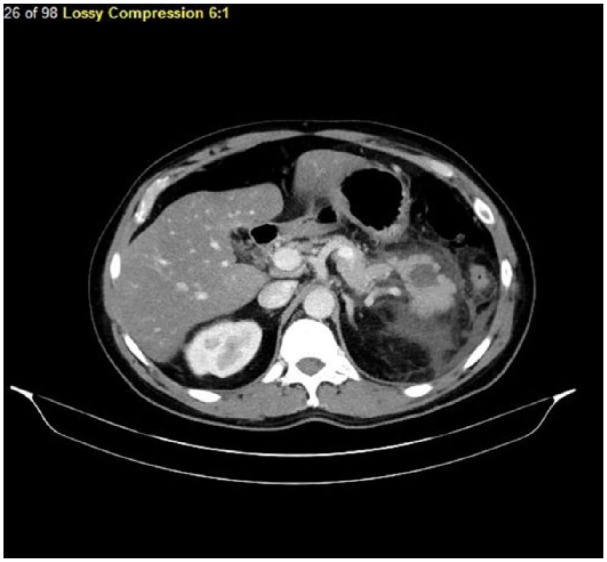
Section of computed tomography scan showing hypodense parenchyma with peripancreatic fluid collections.

**Figure 2.**
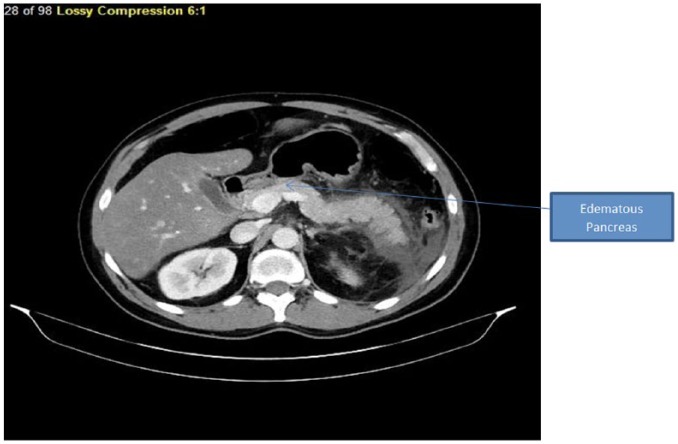
Section of computed tomography scan showing diffuse parenchymal enlargement with retroperitoneal fat stranding.

On the first day of admission, the patient experienced abdominal discomfort that worsened alongside a fever of 101.3°F. His clinical picture began to deteriorate on day 2, with a pulse of 124 beats per minute, and blood pressure of 98/67 mm Hg. Physical examination was notable for mild tenderness in the epigastrium to palpation without distension, organomegaly, or rigidity. Laboratory evaluation showed a hematocrit of 39%, leukocyte 14 200 (4500-11 000 mm^3^) with neutrophil predominance of 85%, platelets 113 000 (130 000-400 000 mm^3^), sodium 122 mEq/L (136-144), creatinine 0.6 (0.4-1.3), C-reactive protein 47 mg/L (0-3 mg/L), amylase 140 U/L (28-100 U/L), and lipase 560 U/L (22-51). The most alarming laboratory finding was a severe elevation of TGs of 10 612 mg/dL (0-149 mg/dL). Liver chemistry was notable for a bilirubin of 2.8 mg/dL, aspartate aminotransferase 90 IU/L (8-46 IU/L), alanine aminotransferase 60 IU/L (7-55 IU/L), alkaline phosphatase 160 IU/L (45-115 IU/L), protein 6.9 (6.1-7.9 g/dL), and prothrombin time 11.6 (9.8-13.4 seconds). Serum immunoglobulin G4 level was 75 mg/dL (8-140 mg/dL). Urinalysis and chest X-ray were unremarkable. Abdominal ultrasound showed a normal gallbladder and liver with normal intrahepatic and extrahepatic bile ducts. CT scan of the abdomen and pelvis showed a hypodense lesion in the pancreas surrounded by a moderate amount of peripancreatic fluid (Figures 1 and 2). Ranson’s score was calculated at 2, indicating mild AP.

Initial management included aggressive intravenous rehydration therapy, antiemetics, and opioids for pain control while awaiting urgent transfer to the intensive care unit for the supportive measure and close monitoring. The patient had a persistent low-grade fever, tachycardia of 112 beats per minute, and elevated lactic acid of 2.7 (0.5-1). Blood cultures were drawn, and meropenem was initiated empirically due to a high clinical suspicion of pancreatic infection pending culture sensitivity. Insulin was initiated in an attempt to reduce TG levels rapidly. Infusion of insulin was initiated at a rate of 1 to 2 U/kg/day with 5% dextrose in 100-mL infusion to prevent hypoglycemia. His blood glucose level was ranging between 180 mg/dL and 210 mg/dL (random = 140-200 mg/dL). He never developed hypoglycemia during the duration of insulin infusion. The decision was made to initiate apheresis, but the patient improved significantly with insulin infusion alone along with supportive measures. There was a fall in TG level to 6120 mg/dL on day 2 after insulin infusion, with a further drop to 3510 mg/dL by day 4, and finally levels decreased to 500 mg/dL by day 7. Insulin was used for the total duration of 8 days until TG level decreased below 300 mg/dL, and unlike diabetic ketoacidosis, the patient was not bridged with subcutaneous insulin therapy. The patient was nil per os (nothing by mouth) initially, and from day 4 of hospitalization, he began tolerating oral feeds in addition to fenofibrate, which was initiated at a dose of 90 mg/day. Empiric antibiotic therapy was stopped due to negative blood cultures. Based on a temporal association as well as ruling out other competing etiologies, a final diagnosis of HTG-induced AP was made. His last TG level recorded was 325 mg/dL during the hospital stay, and the patient was discharged after recovery from AP on long-term fenofibrate therapy. The patient was followed-up after 3 months from the time of discharge with the TG level of 230 mg/dL without any further complications. The patient was counseled to continue fenofibrate indefinitely in order to prevent further attacks of AP.

## Discussion

Acute pancreatitis is defined as inflammation of the pancreas that develops suddenly and can be life-threatening. The incidence of AP in the United States is 40 per 100 000 persons.^2^ AP is the leading cause of admissions to the hospital for gastrointestinal-related disorders in the United States as well as many other countries.^3^ HTG, although rare, is the third leading cause of AP after gallstones and alcohol use, and it can cause up to 7% AP cases. The most important risk factor was TG levels that range to 1000 mg/dL, as in our patient who had a TG level of greater than 10 000 mg/dL.^4,5^

HTG-induced AP most commonly occurs in patients with prior lipid disorders or abnormalities precipitated by a secondary factor such as the use of alcohol, medication, or poorly controlled diabetes. Genetic factors determine more than 60% of the variability in serum lipids, such as patients with type I, III, IV, and V hypolipoproteinemia.^6,7^ It has been shown that patients who have HTG that is either drug-induced or due to diet, without the risk factors of obesity, diabetes, and alcohol, account for only 15% of AP cases associated with HTG.^8^ The family history of HTG-induced AP is an important risk factor; however, our patient had no predisposing genetic risk factors or a known family history.

TG levels greater than 1000 mg/dL are considered severe HTG, and levels over 2000 mg/dL are considered very severe HTG and warrant emergent reduction.^9^ The pathogenesis of HTG-induced pancreatitis is unclear; it is thought to result from toxic injury to acinar cells and capillary endothelium. The hydrolysis of TGs by pancreatic lipase and release of free fatty acids (FFAs) induce free radical damage, which can directly injure cell membranes.^10^ Additionally, severe or very severe HTG along with high lipase levels (>3 times the upper limit of normal) are associated with very high FFA levels and can further be complicated by systemic inflammation from AP, direct activation of toll-like receptor 2 and toll-like receptor 4 by FFA, and direct lipotoxicity.^11,12^

The level of HTG is essential in the management, as there are no definite guidelines for treatment solely based on the TG level. The use of insulin, heparin, and plasmapheresis are active treatment modalities that have been used along with symptomatic management with pain control, intravenous fluids, and bowel rest. The use of heparin remains controversial; studies have shown it to stimulate the release of lipoprotein lipase from endothelial cells, allowing it to degrade chylomicrons thus decreasing TG levels.^13^ Plasmapheresis can be used for removal of plasma lipoproteins, reducing TG levels rapidly in case of organ dysfunction or failure.^14^ A few case reports in the literature have demonstrated that apheresis for HTG-induced AP must be initiated early for benefit. Apheresis may be particularly important for the treatment of hypertriglyceridemic necrotizing pancreatitis immediately after its onset.^15^ Our patient did not develop necrotizing pancreatitis and showed improvement with insulin therapy alone. Stefanutti et al reported a case with an HTG level of 11355 mg/dL, which improved, with early initiation of plasmapheresis while the patient was in the emergency department. In this acute case, insulin was not administered.^16^ Our patient had a very high HTG level (>10 000 mg/dL), and he improved significantly with insulin infusion alone. Aryal et al described a similar patient who showed improvement with insulin therapy with TG level as high as 15 215 mg/dL.^17^ In our case, we initiated insulin infusion with an eventual reduction in TG level to 500 mg/dL over a 7-day period. The decrease in TG level took slightly longer compared with other published literature (Table 1), where the use of insulin as a sole therapy to lower TG levels to less than 500 mg/dL was achieved over a period of 3.5 to 4 days.^18-20^ However, unlike other published case reports where TG levels in the majority of patients were below 10 000 mg/dL, our case had a TG greater than 10 000 mg/dL, which may have contributed to the delayed fall of TG below 500 mg/dL when treatment was initiated. The mortality benefit of insulin and apheresis remains unclear, as illustrated in a case report by Melnick et al, where the patient expired despite the use of insulin and apheresis, which may have been attributable to the severe onset of AP. It should be noted that apheresis was delayed in use, in this case, having been initiated 3 weeks after the onset of AP.^21^ True efficacy of plasmapheresis is unknown as there are no randomized controlled trials; therefore, definitive conclusions on the efficacy of apheresis in reducing AP severity cannot be made.^22^ Furthermore, due to its lack of availability at times, risks, and expense, insulin can be used as an alternative and potentially safer method of treatment. There remains a paucity of established guidelines for HTG-induced AP management in the acute setting. However, long-term management strategies to prevent recurrent AP secondary to HTG have been established. An example of a long-term maintenance therapy is the use of fibrates, which not only reduce serum TG levels by 50% but also increase high-density lipoprotein by 20%.^23^ Fibrates decrease hepatic secretion of very-low-density lipoprotein and increase lipolysis of TGs by regulating a specific receptor in the liver, peroxisome proliferator-activated receptors-α.^24^ Our patient was managed with fibrates as outpatient therapy on long-term maintenance care. Lifestyle modifications such as weight loss in obese patients and aerobic exercise should be performed. Alcohol and concentrated sugars should be avoided, and strict glycemic control in diabetics should be the first-line therapy.^25^ Other risk factors for the development of AP such as smoking should be avoided too.^25,26^ Additional novel modalities to reduce TG levels include the use of statin therapy, which decreases cholesterol levels, and omega-3 fatty acids, which lower TG levels by 20% when used in conjunction with statins.^27^ Antioxidant therapies with vitamins such as vitamin C and α-tocopherol have been demonstrated to protect the acinar cells of the pancreas from free radical damage. These therapies have been used in recurrent AP cases due to refractory HTG after medical treatment.^28^

**Table 1.** Case Reports Showing Improvement in Triglyceride Levels After Insulin Infusion ± Additional Treatment Modalities.

Case Reports	HTG-Induced AP	Treatment Modalities	Outcome
Aryal et al^17^	15 215 mg/dL	Insulin and heparin infusions	Triglyceride improved to 363 mg/dL on day 6.
Melnick et al^21^	>10 000 mg/dL	Insulin followed by plasmapheresis	Required plasmapheresis after day 5 on insulin owing to triglyceride 6069 mg/dL and worsening symptoms.
Khan et al	3525 mg/dL	Insulin infusion only	Improved on next day with triglyceride 973 mg/dL.
Present case report	10 612 mg/dL	Insulin infusion only	TG decreased below 500 mg/dL on day 6.

Abbreviations: HTG, hypertriglyceridemia; AP, acute pancreatitis.

## Conclusion

There are no current established guidelines for the treatment of very severe HTG-induced AP, although insulin, heparin, and plasmapheresis have all been used in the literature. The unique feature of our case can be emphasized with the quick and effective response to insulin therapy alone. Additionally, the cost-effectiveness of plasmapheresis remains uncertain, and further investigations are warranted to establish best-care practices.
